# A Longitudinal Study of Symptoms of Oropharyngeal Dysphagia in an Elderly Community-Dwelling Population

**DOI:** 10.1007/s00455-016-9715-9

**Published:** 2016-06-15

**Authors:** Danielle Nimmons, Emilia Michou, Maureen Jones, Neil Pendleton, Michael Horan, Shaheen Hamdy

**Affiliations:** Faculty of Medical and Human Sciences, Centre for Gastrointestinal Sciences, Institute of Inflammation and Repair, University of Manchester, Salford Royal NHS Foundation Trust, Clinical Sciences Building, Manchester, M6 8HD UK; Centre for Clinical and Cognitive Neuroscience, Institute of Brain, Behaviour and Mental Health, University of Manchester, Manchester, UK; Centre for Integrated Genomic Medical Research, Manchester, UK; Salford Royal NHS Foundation Trust, Salford, UK; Manchester Medical School, University of Manchester, Manchester, UK

**Keywords:** Sydney Swallow Questionnaire, Older people, Deglutition, Deglutition disorders

## Abstract

Dysphagia has been estimated to affect around 8–16 % of healthy elderly individuals living in the community. The present study investigated the stability of perceived dysphagia symptoms over a 3-year period and whether such symptoms predicted death outcomes. A population of 800 and 550 elderly community-dwelling individuals were sent the Sydney Swallow Questionnaire (SSQ) in 2009 and 2012, respectively, where an arbitrary score of 180 or more was chosen to indicate symptomatic dysphagia. The telephone interview cognitive screen measured cognitive performance and the Geriatric Depression Scale measured depression. Regression models were used to investigate associations with dysphagia symptom scores, cognition, depression, age, gender and a history of stroke; a paired *t* test was used to examine if individual mean scores had changed. A total of 528 participants were included in the analysis. In 2009, dysphagia was associated with age (*P* = 0.028, OR 1.07, CI 1.01, 1.13) and stroke (*P* = 0.046, OR 2.04, CI 1.01, 4.11) but these associations were no longer present in 2012. Those who had symptomatic dysphagia in 2009 (*n* = 75) showed a shift towards improvement in swallowing (*P* < 0.001, mean = −174.4, CI −243.6, −105.3), and for those who died from pneumonia, there was no association between the SSQ derived swallowing score and death (*P* = 0.509, OR 0.10, CI −0.41, −0.20). We conclude that swallowing symptoms are a temporally dynamic process, which increases our knowledge on swallowing in the elderly.

## Introduction

Oropharyngeal dysphagia (OD) can be considered a major debilitating symptom often associated with co-morbid diseases, more common with increasing age . The prevalence of dysphagia in community-dwelling elderly populations has been reported to be between 8 and 16 % [1–6]. Although no studies have longitudinally investigated dysphagia in this population, it has been shown that 12 months after stroke swallowing usually improves, mainly between 3 and 6 months [7]. Also, the association between dysphagia and death has not been studied in elderly individuals living in the community but it is known that dysphagia can result in malnutrition and reduced nutritional status, increasing the risk of pneumonia, a common cause of mortality in patients with dementia and other cognitive disorders [8, 9]. The literature suggests relationships between dysphagia in the elderly and independent variables, such as age, depression and cognition [4, 10–12], but few include community-dwelling older people [4].

The Sydney Swallow Questionnaire (SSQ) is a self-report inventory used to measure oropharyngeal dysphagia. Several studies have demonstrated its strong content, construct, discriminant and predictive validity and test–retest reliability in a range of different populations, for example, in head and neck cancer patients and in patients with Duchenne muscular dystrophy [13–15]. It should be recognised, however, that the methods used were not uniform across all studies and participant numbers varied.

The aim of this study was to therefore determine, in an elderly cohort living in the community, if perceived dysphagia symptoms as assessed by SSQ over a 3-year period would remain stable or deteriorate. A second aim was to investigate whether the presence of dysphagia symptoms determined by SSQ was a predictor of death-related outcomes. Our hypotheses were that dysphagia symptoms would worsen over the 3-year period, as the population aged and that the presence of dysphagic symptoms would be positively associated with an increased likelihood of death during those 3 years.

## Methods

### Study Population and Study Design

The study cohort was composed of 800 elderly community-dwelling individuals who were all surviving participants in the University of Manchester Longitudinal Study of cognition during ageing [16], which started in 1983. This study was designed to observe changes in cognitive function in over 6000 volunteers aged 50 and over. The Sydney Swallow Questionnaire (SSQ) [17] was sent via mail to all 800 members of this study cohort in 2009 and to the surviving 550 members of the same cohort in 2012. Dementia was an exclusion criterion at the time of recruitment. Our study was approved by the local ethics committee (08/H1016/40) and carried out in accordance with the Declaration of Helsinki.

### Sydney Swallow Questionnaire (SSQ)

The Sydney Swallow Questionnaire [17] includes 17 questions related to swallowing function and difficulties, of which 16 questions use an analogue scale for participants to enter their responses. The scale is composed of a 105 mm (mm) continuous line that follows each question and the participants’ score can be translated between 0 and 100 for each of the 16 questions. On either end of the line are opposing statements regarding specific impairments of oropharyngeal swallowing. The questionnaire includes instructions asking participants to place an “X” at a certain distance along the line to indicate the magnitude of their response. The distance to the centre of the mark can be measured to the nearest mm and made into a score out of 100 for each question. An example of a question is: ‘How much difficulty do you have in swallowing at present?’. A mark placed in the first 5 mm of the line is scored as 0 for that question. For question 12, instead of a visual analogue scale, the scores can be 0, 20, 40, 60, 80 or 100 depending on the participant’s answer. The maximum possible total score is 1700 and a higher score indicates greater perceived swallowing problems.

### Cognitive Tests and Depression Assessment

The telephone interview cognitive screen (TICS-m) is a short screening tool including a word list learning task and was used to measure cognitive performance [18]. It is a 13-item test with scores ranging from 0 to 50 and tests orientation, concentration, immediate and delayed memory, naming, calculation, comprehension and reasoning. This was done over the phone and a score below 22 was used to define ‘cognitive impairment’ [18] and warranted a home visit to further test cognition. The Geriatric Depression Scale (GDS) questionnaire was used to measure depression [19]. The maximum possible score on the questionnaire was 15 and a score of 5 or above indicated depressive tendency. Both TICS-m and GDS tests were conducted on participants between 24 and 36 months apart, once during the period 2009–2010 and then again in 2011–2012.

### Mortality Data

As part of approved user agreement the Health and Social Care Information Centre (HSCIC) provides date and cause of death of volunteers on an interval basis, which is then incorporated into an anonymised data set including the University of Manchester Longitudinal Study of cognition during ageing. The office of national statistics was the provider until this function was transferred to HSCIC.

### Statistical Analysis

All data were analysed using SPSS version 20.0 software (SPSS Inc., Chicago, IL, USA). A score of 180 in SSQ was used as the threshold for (significant) symptomatic dysphagia, as per results from a previous validation study for the questionnaire. This showed strong face, content, construct validity and test–retest reliability for the SSQ in a population with neuromyogenic dysphagia, where videofluoroscopy was included to examine swallowing [17].

Regression models were used to investigate associations with swallowing function. Using results from 2009 to 2012, logistic regression models were used and dysphagia (a score of 180 or more) was used as the dependent variable. For those who had died from pneumonia, a linear regression model was calculated to investigate association with total score on questionnaire in 2009. The following independent variables were accounted for age, gender, cognition and depression levels as per scores in the assessments. Stroke and Parkinson’s disease are co-morbidities in older people that are important in relation to dysphagia and were included as confounding variables in these regression models depending on their prevalence in the study population. A *P* value of 0.05 or less was applied to represent significance.

Differences in swallow score for participants between 2009 and 2012 were normally distributed; hence a parametric paired *t* test was used to examine if individual mean score had changed in this time for all of the participants. By contrast, when participants were separated into two groups according to whether they had a total swallow score of 180 or more, or a score of less than 180, a non-parametric Wilcoxon signed rank test was applied as these data were not normally distributed. Categorical data are presented as numbers of subjects and percentages. Continuous data are presented using mean and standard deviation if normally distributed, or median and interquartile range if skewed.

## Results

### Participant Characteristics

In 2009, 634/800 participants and 467/550 participants in 2012 successfully completed the SSQ, making the response rate in those years 80 and 85 %, respectively. On entry into the study, 52 (8 %) participants had a history of stroke and 6 (1 %) Parkinson’s disease, thus stroke was included as a confounding variable in the analysis. Four hundred and twenty-nine participants had completed the questionnaire both in 2009 and 2012, and 99 participants had died since completing the questionnaire in 2009. This meant that there were 106 participants who had completed the questionnaire in 2009 but did not in 2012, although they were still alive. The characteristics for the different participant groups are shown in Table 1 and include 2009 results for the following groups:

**Table 1:** Male/female ratio, mean age and median SSQ swallow scores in 2009 for Groups A, participants who completed the questionnaire in 2009 and 2012; Group B, those who later died between 2009 and 2012 and Group C, those who did not complete the questionnaire in 2012 but did not die in this time period

Participant group	Number	Male:female ratio	Mean age (standard deviation)	Median swallow score	Interquartile range	Range
A	429	25:75	81 (5.0)	40	20,107	0–894
B	99	33:67	85 (5.1)	42	20,116	0–687
C	106	19:81	83 (5.2)	40	20,114	0–763

- Group A—participants who completed the questionnaire in 2009 and 2012;
- Group B—those who later died between 2009 and 2012 and
- Group C—those who did not complete the questionnaire in 2012 but did not die in this time period.

Those who had completed the questionnaire both in 2009 and 2012 (Group A), and those who had died since completing the questionnaire in 2009 (Group B) were included in the longitudinal analysis.

**Fig. 1:**
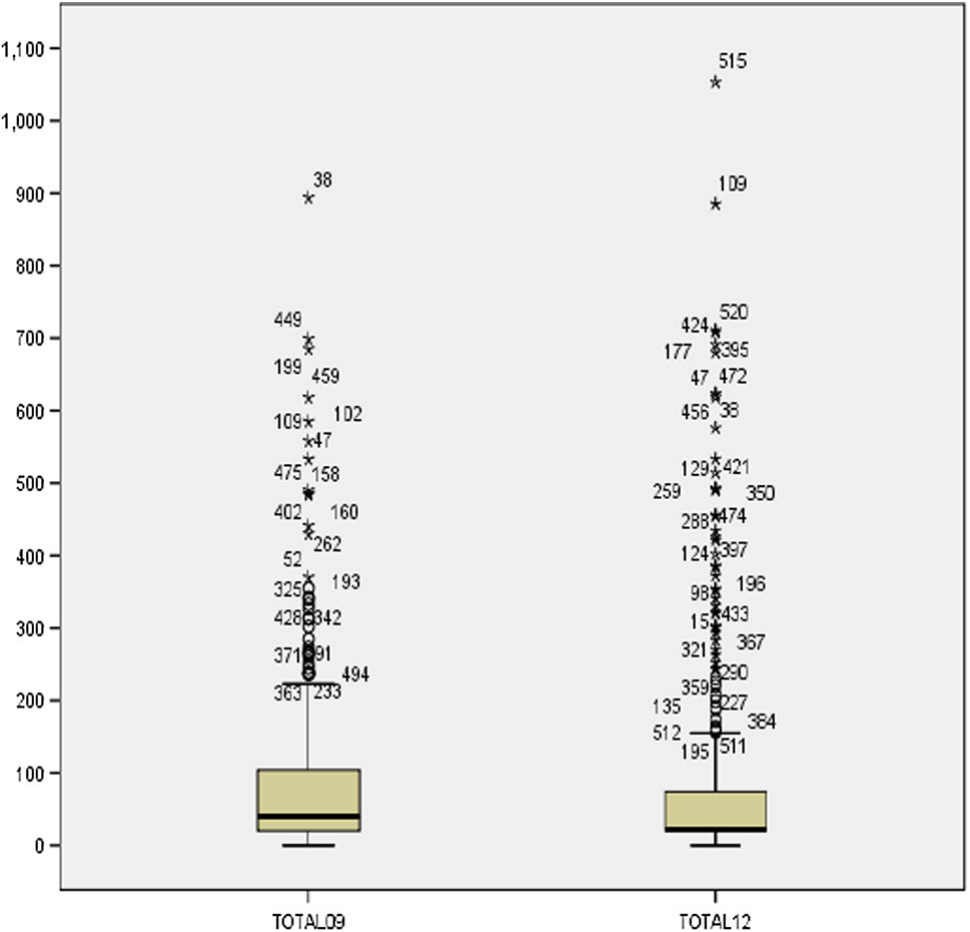
*Box* and *whisker plot* showing the distribution of the total scores attained on the questionnaire in 2009 and 2012 for Group A (*n* = 429)

Figure 1 shows the distribution of the total scores attained on the questionnaire in 2009 and 2012 for Group A and Table 2 shows their characteristics in both years. Figure 1 and Table 2 show that along with the median swallow score, the interquartile range also reduced over the 3-year period, despite the mean age increasing from 81 to 85 years old. The change in median score was found to be statistically significant (*P* = 0.009).

**Table 2:** Male/female ratio, mean age and median SDQ swallow scores for Groups A (participants who completed the questionnaire in 2009 and 2012) in 2009 and 2012

Year	Male:female ratio	Mean age (standard deviation)	Median swallow score	Interquartile range	Range
2009	25:75	81 (5.0)	40	20,107	0–894
2012	22:78	85 (5.1)	22	2074	0–1053

### Swallowing, TICS-m and GDS Profiles in 2009 for Group A&B and 2012 for Group A

**Fig. 2:**
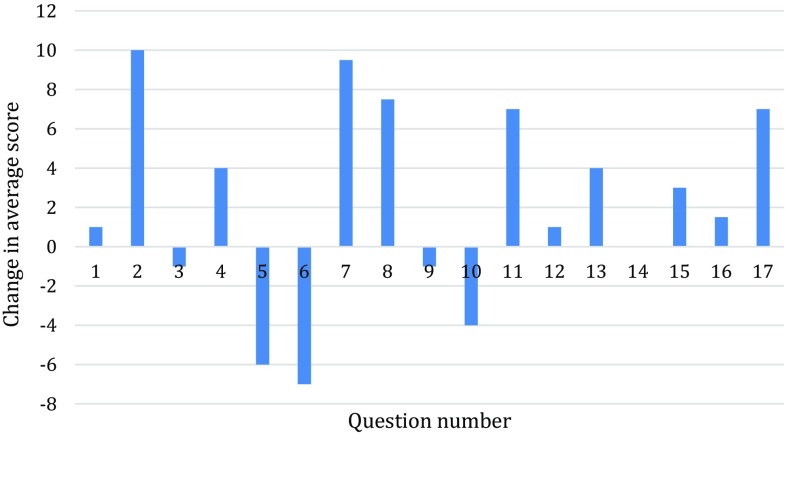
*Bar chart* showing change in average score for each of the 17 questions of the SSQ from 2009 to 2012

With the use of the dysphagia cut-off score of 180 to indicate perceived swallowing difficulties, the prevalence of dysphagia in this elderly population was 14.2 % in 2009 and 13.4 % in 2012. Changes in median score for each question ranged from −7 to +10, shown in Fig. 2. In both years, the three commonest symptoms in this group were the following:

- A feeling of food getting stuck in the throat (Q9).
- Difficulty swallowing hard foods at present (Q5).
- A sensation of choking/coughing on swallowing (Q10).

The mean TICS-m scores assessing cognitive performance were 29.9 and 30.1 in 2009 and 2012, respectively, with a standard deviation of 3.9 in both years. Only 1.5 % of the population in 2009 and 2012 had a score of 22 or less, indicating mild cognitive impairment. In both years, the median GDS across the population was 1 and scores ranged from 0 to 12. In 2009 and 2012, 5.3 and 5.5 % of respondents had a score of 5 or more indicating signs of depression or unhappiness.

### Clinico-Demographic Associations with Dysphagia Score in 2009 and 2012

It was investigated whether dysphagia was significantly associated with the chosen variables stated above. In 2009, significant associations were found between symptomatic dysphagia and age (*P* = 0.028, OR 1.07, CI 1.01, 1.13) and stroke (*P* = 0.046, OR 2.04, CI 1.01, 4.11). No association was found with gender (*P* = 0.229, OR 1.55, CI 0.76, 3.19), TICS-m (*P* = 0.342, OR 1.04, CI 0.96, 1.11) and GDS (*P* = 0.235, OR 1.09, CI 0.94, 1.26).

However, in 2012, no significant association was found between symptomatic dysphagia and age (*P* = 0.890, OR 1.00, CI 0.94, 1.047), gender (*P* = 0.704, OR 1.16, CI 0.53, 2.53), GDS (*P* = 0.966, OR 0.98, CI 0.36, 2.64), TICS-m (*P* = 0.446, OR 0.44, CI 0.05, 3.56) and stroke (*P* = 0.097, OR 2.28, CI 0.86, 6.03). The confidence intervals for each variable comfortably included 1, which shows that these variables do not have a significant direct link on developing dysphagia.

### Comparison Between the 2009 and 2012 Results

The distribution of the change in the total score from 2009 to 2012 was found to be normal and thus a paired sample t-test was conducted to compare these results. The mean total scores seen between the two (complete) sets of data were unchanged (mean difference = −3.4, CI −18.5, 11.6, *P* = 0.655), signifying that over the 3-year period participants felt their swallowing did not change much.

The population was then divided into two groups, those who had a total score of 180 or more, or a score of less than 180 in 2009, i.e. those who did and did not have dysphagia in 2009. There was a mean reduction in total SSQ for those who had a score of 180 or more in 2009 (mean difference = −174.4, CI −243.6, −105.3). The distributions of these data were not normal so the non-parametric Wilcoxon signed rank test was performed, which was found to be statistically significant (*P* < 0.001). This result suggests that those who had dysphagia in 2009 experienced a substantial shift towards improvement in their swallowing. However, those who had a score of less than 180 showed some stability over time, with a change score from 2009 to 2012 (mean increase = 20.7, CI 8.2–33.2) that was not significant using the Wilcoxon Signed Rank Test (*P* = 0.859).

### Death-Related Outcomes

In 2012, ninety-nine participants (19 %) had died having completed the questionnaire in 2009 (Group B). The median total SSQ score in 2009 for those who later died was 42. By comparison, the median total score in 2009 for those who completed the questionnaire in 2012 was 40 (Group A). The interquartile range for both groups was also similar (Table 1).

Of those who died, 19.6 % had a score of 180 or more in 2009, indicating dysphagia. In comparison, for those who completed the questionnaire in both 2009 and 2012, 12.0 % experienced swallowing impairments in 2009. Furthermore, a greater proportion of participants who later died had cognitive impairment (TICS score of less than 22) in 2009 compared to those who did not, being 3.3 and 1.4 %, respectively.

For those who died, 16 (16 %) participants had pneumonia as a cause of death on death certificate. For 14 of these participants, pneumonia was the direct cause of death and for 2 it was an underlying condition leading to another cause of death.

Results were then compared for those who had pneumonia as a cause of death on the death certificate and those who died from other causes. The median total score for those who had died from pneumonia was 50; by contrast this was 41.5 for those who had died of other causes (Table 3). Those who died from pneumonia had a greater median total score indicating that these participants on average perceived their swallowing to be worse in 2009, compared to those who had died from another illness. Finally, for those who died from pneumonia, no significant association was found between the 2009 total questionnaire score, the time between the questionnaire being sent out and death (*P* = 0.509, OR 0.10, CI −0.41, −0.20). This linear regression model thus indicates that perceived symptomatic dysphagia does not predict pneumonia and subsequent death from the illness.

**Table 3:** Comparison of data between those who died from pneumonia having completed the questionnaire in 2009 and those who had died but not from pneumonia

Death type	Number	Median	IQR
Non-pneumonia	83	41.5	20,142.75
Pneumonia	16	50	20,82.75

## Discussion

Dysphagia has been well described in acutely ill patients and in those affected by neurological disease but less is known about how it affects relatively independent community-dwelling elderly individuals. To the best of our knowledge, this study was the first of its kind to use the SSQ for longitudinal analysis and observe how participants perceive their swallowing over a period of years.

Surprisingly, and contrary to our hypothesis, as people aged their swallowing was not being reported to be significantly declining. In fact, those who scored high in the SSQ in 2009 showed a clear shift towards improvement, which at first glance seems counter-intuitive. However, there could be a number of explanations for such an observation.

Ageing has been associated with increased risk for swallowing disorders due to age-related changes, such as sarcopenia, sensory problems and muscle weakness [20]. However, direct and causal relationships between the several factors and swallowing disorders have as of yet not been found. On the other hand, the neural processes controlling swallowing in older people have been investigated and while there have been differing results [21, 22], there is some suggestion that brain compensation might take place. Indeed, compensation in function is thought to occur in older people with no neurological lesions [23], although the evidence for this phenomenon in swallowing function is less clear. Compensation is a form of brain plasticity, producing short and long lasting changes in brain properties that can be morphological or functional [24]. It allows the nervous system to adapt and restructure itself throughout life. We have previously observed recovery of swallowing function in stroke, which is usually countered in aged populations. This recovery in function was correlated to plastic changes on cortical levels [25]. In our study, these compensatory plastic changes could have taken place in the participants who had symptomatic dysphagia in 2009, while their nervous system adapted to changes to the swallowing mechanism with increasing age. However, it is worth noting that participants were not exposed to interventions to improve dysphagia.

Potentially, an alternative explanation could be that the perceived improvement may also be due to chance variation in symptoms experienced by participants over the 3-year period leading to regression towards the mean, a phenomenon where the second measurement of a variable is closer to the mean if the first measurement is more extreme [26]. In addition, participants could have become more familiar in their understanding of the questions or even awareness of swallowing difficulties could have added to being familiar with the questions. Although the SSQ has shown strong face, content, construct validity and test–retest reliability [17]; there is a lack of longitudinal validity for this questionnaire.

Of interest, no significant association was found between total questionnaire scores in 2012 and any of the independent variables tested: age, gender, depression, stroke and cognition. This was surprising given that in our study stroke and age were found to be associated with dysphagia in 2009, and both age and depression were found to be significantly associated with dysphagia score in the same study population when the questionnaire was mailed in 2009 [4]. This could be explained by either a different cut-off used or a non-linear relationship between age and dysphagia (and other co-morbidities). Those who experience dysphagia have more of a decline in swallowing at a younger age, compared to when they are older and experience less of a decline in swallowing, leading to a plateau effect. Hence, at the extremes of age, in a homogeneous older population, the effect of age may be lost. Depression was no longer associated with dysphagia when stroke was added as a variable, indicating a possible interaction between stroke and depression. Post-stroke depression has been described as an important complication of stroke [27] and this possible interaction could be examined in further studies.

This study adds to current knowledge regarding the changes in swallowing function and more specifically in those who are described as ‘the oldest old’. Understanding such changes is becoming more and more important with an increasingly ageing population globally. Ultimately, this may contribute to advancements in dysphagia management, which remains limited [28]. Our study did utilise the SSQ, which is a subjective measure of swallowing function. Using such a questionnaire could be introduced into common practice as an indicator for swallowing problems and serve as a central tool in enabling clinicians to understand how patients perceive their own health problems. It has been suggested that the questionnaire needs psychometric reassessment, and if overall methodological quality shows improvement, its use in daily clinics and research can be justified [29]. However, it may be that it can only be used as an indicator and should be used in conjunction with objective tools of assessment. And although a recent study investigating SSQ in a non-dysphagic cohort demonstrated an upper limit of normal that was not affected by age or gender [30], it seems likely that such questionnaires will have to be used in caution in certain groups of people, for example those with cognitive decline.

Several limitations of the present study should be noted. To the best of our knowledge, the study cohort was independent and assumed “healthy” for their age; however, it is possible that participants were suffering from illness when they completed the questionnaire and that this could have affected their swallowing. One hundred and six participants completed SSQ in 2009 but not in 2012 and we do not know why; reasons could include new morbidity not allowing them to complete it, which may have affected the results. Moreover, the questionnaire was used as a proxy for measuring swallowing function and was the best tool available at the time, and it should be acknowledged that it may not be the most accurate or stable tool for longitudinal swallowing assessment self-reporting. Reliability of the questionnaire used on multiple occasions could not be tested due to a 3-year interval between the SSQ being administered. No gold standard could be applied to assess swallowing function (safety and efficiency), such as videofluoroscopy, so a comparison of sensitivity or specificity of these methods for measuring subjective swallowing function was not possible. This study also demonstrates the difficulty in using questionnaires to measure something that is inherently subjective. This is made even more difficult when there lacks a universal definition for ageing. Chronological age is used to define who is ‘old’ and this is used as an indication for illness. A better definition of ageing would thus improve future studies in the area and its measurement. Finally, death certificates were used in analysis, which are regarded as important sources of data for death mortality. However, it is well documented that death certificates lack accuracy and there are discrepancies between death certificates and autopsy results [31]. Thus, without autopsy we are unable to know the true reason of death for participants who died during the study period and if they were directly related to dysphagia.

In conclusion, swallowing symptomatology is a dynamic process and perceived swallowing dysfunction did not predict death in this cohort. Also, our study demonstrates that swallowing questionnaires may not be as stable over time as expected.
